# LONG-TERM AND INTERGENERATIONAL EFFECTS OF EARLY-LIFE EXPOSURE TO THE 1959–1961 CHINESE FAMINE ON HEALTH

**DOI:** 10.1093/geroni/igae098.2152

**Published:** 2024-12-31

**Authors:** Mengling Cheng, Nicolas Sommet, Marko Kerac, Dario Spini

**Affiliations:** University of Lausanne / East China University of Science and Technology, Switzerland; University of Lausanne, Lausanne, Vaud, Switzerland; London School of Hygiene & Tropical Medicine, London, England, United Kingdom; University of Lausanne, Lausanne, Vaud, Switzerland

## Abstract

Undernutrition in early life is associated with increased risk of poor health and mortality in later life. Less is known if the health effects of early-life undernutrition transmit across generations. This study examined the associations between early-life famine exposure and self-rated health in the first generation (F1) and the subsequent generation (F2). We used data from the China Health and Retirement Longitudinal Study (2011-2020, 11,000 participants). We classified F1 participants by famine exposure (exposed/unexposed) and timing at famine exposure (exposed in utero, infancy, pre-school, primary school, or adolescence); we classified F2 offspring by their parents’ famine exposure status. We performed growth curve models to investigate the associations between early-life famine exposure and self-rated health in F1 and F2. The first generation (F1) exposed to famine in utero, infancy, pre-school, primary school, or adolescence had poorer self-rated health than those unexposed (IRR=1.06, 95% CI [1.04 – 1.07], p <.001). The offspring generation (F2) whose parents were exposed to famine also had poorer self-rated health than those of unexposed parents (IRR=1.05, 95% CI [1.01 – 1.10], p <.05). Findings of this study show the long-term and intergenerational effects of early-life famine exposure on health, indicating cumulative advantage and disadvantage over the life course and across generations. Our study highlights the need to prevent and treat child/adolescent undernutrition to improve health in the long run.

